# PERK Signaling Pathway Mediates the Hepatoprotective Effects of Naringenin Against Cadmium‐Induced Liver Injury in Rats

**DOI:** 10.1002/jbt.71044

**Published:** 2026-07-22

**Authors:** Chengxiang Guo, Hao Ling, Mengmeng Gao, Yinan Hu, Jing Zhu, Jicang Wang

**Affiliations:** ^1^ College of Animal Science and Technology Henan University of Science and Technology Luoyang PR China

**Keywords:** cadmium, endoplasmic reticulum stress, liver, naringenin, PERK

## Abstract

Cadmium (Cd), a heavy metal element with extensive industrial applications, exhibits significant toxicological risks in biological systems (Zhu et al. 2019). Naringenin (Nar), a bioactive flavonoid possessing potent antioxidant properties, demonstrates ameliorative effects against Cd toxicity. 24 male SD rats were randomly allocated into four groups: Control group; Cd group; Cd + Nar group; Nar group. Determination of biochemical indicators, antioxidant indicators; reverse transcription quantitative real‐time PCR (RT‐qPCR) and Western blot were used to detect the expression levels of related mRNA and protein. Cd exposure reduced rat body weight and increased liver‐to‐body weight ratio, along with elevations in ALT, AST, GSH, and MDA. Conversely, Cd + Nar treatment increased body weight, reduced liver organ coefficient, and decreased these biomarkers. HE staining revealed expanded hepatic sinusoids in the Cd group. RT‐qPCR and Western blot showed Cd upregulated GRP78, PERK, and CHOP expression at both mRNA and protein levels, while Cd + Nar treatment attenuated these increases. Cd activates the PERK signaling pathway through ERS and causes liver damage. Nar can reduce liver toxicity. The mechanism may be related to the inhibition of PERK activation by Nar.

## Graphical abstract

On the basis of establishing an experimental animal model of cadmium poisoning, the role of PERK pathway in cadmium‐induced liver injury in rats and the protective effect of naringenin were detected. It is of great scientific significance and application value to explore the protective effect and mechanism of PERK signaling pathway naringenin on cadmium‐induced liver injury in rats.

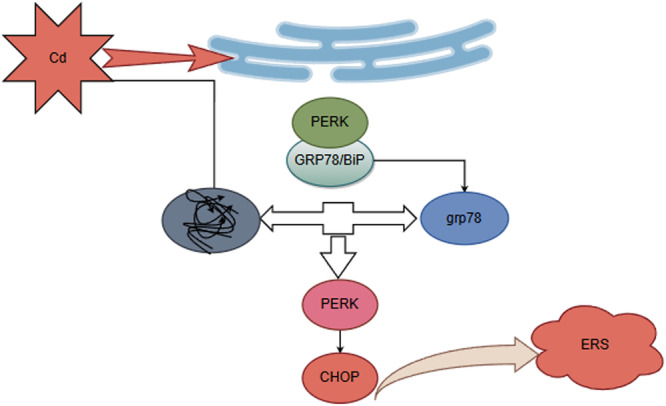

## 1 Introduction

Cadmium (Cd), a toxic heavy metal posing profound threats to human and animal health, is widely distributed across various sources including industrial production and environmental pollution. It can be inhaled via the respiratory tract, where Cd‐containing fine particles insidiously enter the lungs and gradually deposit during the routine respiration of humans and animals; it can also enter the human digestive system through the food chain, migrating through from soil to crops and ultimately into the diet. Once inside the human body, Cd continuously accumulates in various tissues due to its long biological half‐life, rendering its effective elimination by the body extremely slow. This significantly increases the likelihood of sustained harm from Cd in vivo. As a vital metabolic organ, the liver is the primary target of Cd‐induced damage, exhibiting pronounced hepatotoxicity [1, 2].

Cd toxicity was exhibited a dose‐dependent relationship with dose [3]. When Cd reaches a certain threshold in the body, its toxic effects are initiated. The cell membrane, as a critical barrier of the cell, is the first to be impacted. Cd disrupts ion channels and transport proteins on the cell membrane, impairing the normal substance transport function and causing imbalance in intracellular‐extracellular material exchange [4]. These alterations in membrane structure and function are closely linked to lipid peroxidation. Stimulated by Cd, reactive oxygen species (ROS) increase dramatically, triggering an oxidative stress response. Due to the liver's heavy metabolic workload, its redox balance is particularly vulnerable to disruption, making the liver the most significantly affected organ by Cd‐induced oxidative damage among all tissues [5]. The endoplasmic reticulum (ER) plays a pivotal role in cellular physiology as the primary site for protein folding [6]. Only properly folded proteins can form appropriate spatial conformations and subsequently exert their biological functions [7]. However, the presence of Cd may interfere with the ER's normal physiological processes, compromising protein folding efficiency and quality, and exerting profound effects on overall cellular function and homeostasis [8, 9].

Naringenin (Nar), a predominant natural flavonoid, serves as a central scaffold. As the aglycone of naringin [10], it possesses multiple biological activities inherent to polyphenolic substances, endowing it with unique value in addressing numerous health issues—particularly in counteracting toxic damage induced by xenobiotics such as Cd.

Nar demonstrates robust antioxidative efficacy. Oxidative stress, ubiquitously implicated in biological processes, is recognized by the scientific community as a principal etiological driver for diverse pathophysiological conditions, especially those closely associated with heavy metal poisoning. When the body is exposed to heavy metals like Cd, an overwhelming production of free radicals occurs, which relentlessly attack various biological macromolecules, triggering deleterious reactions such as lipid peroxidation and severely disrupting normal cellular structure and function. Nar combats oxidative stress crises through multiple interconnected pathways: on one hand, it efficiently neutralizes free radicals by rapidly trapping highly oxidizing species and rendering them inactive, thereby blocking further cellular damage; on the other hand, it potently inhibits the lipid peroxidation process to prevent free radical‐mediated peroxidation of membrane lipids and maintain the integrity and stability of biological membranes; collectively reducing oxidative damage through these concerted pathways [11]. Studies in rats have demonstrated that Nar markedly attenuates Cd‐induced hepatotoxicity, alleviating ERS and thereby attenuating the adverse effects of Cd on rat livers [12].

As an ER‐resident type I transmembrane serine/threonine‐protein kinase and prototypic member of the eIF2α kinase family [13, 14], PERK functions as the canonical stress transducer in the unfolded protein response (UPR) pathway [8, 15]. Under proteotoxic stress conditions marked by misfolded protein accumulation [16], the central ER chaperone GRP78 [17] exhibits binding affinity toward misfolded client proteins through its substrate‐binding domain, initiating quality control processes while disengaging from luminal stress sensors. This chaperone redistribution event enables PERK dimerization and autophosphorylation through inter‐subunit kinase domain transactivation, converting the stress transducer to its catalytically competent state [18]. The activated PERK catalytic domain specifically phosphorylates eIF2α at Ser51, inducing phosphorylation‐dependent inhibition of ternary complex formation and subsequent translation initiation suppression. This early‐phase adaptive response reduces nascent protein flux into the ER lumen, thereby mitigating proteostatic imbalance during acute stress challenges. Chronic ER stress provokes maladaptive PERK signaling through sustained eIF2α phosphorylation, which activates the ATF4‐mediated transcriptional program culminating in CHOP overexpression ‐ a terminal effector that promotes hepatocyte apoptosis via Bcl‐2 family protein dysregulation and mitochondrial permeabilization.

Environmental toxicant exposure has emerged as a critical global health challenge affecting both veterinary species and human populations. Of particular concern is Cd contamination ‐ a persistent environmental toxicant with properties in the food chain [4]. Cd demonstrates well‐documented hepatotoxic properties through oxidative stress induction and organelle dysfunction [19]. Nar, a bioactive citrus flavonoid, has demonstrated significant antioxidant and cytoprotective capacities via free radical scavenging and redox homeostasis modulation [20]. This study elucidates the hepatoprotective mechanisms of Nar through PERK‐mediated UPR modulation, providing novel therapeutic strategies for Cd‐induced hepatic damage with translational potential in veterinary and human medicine.

## 2 Materials and Methods

### 2.1 Experimental Reagents

Cd chloride (CdCl_2_; ≥99.95% purity, CAS 7790‐78‐5, MW 228.36) and Nar (≥97% purity, CAS 67604‐48‐2, MW 272.26). Commercial assay kits for biochemical analysis were procured as follows: AST (Cat# C010‐2‐1), ALT (Cat# C009‐2‐1), reduced glutathione (GSH, Cat# A006‐2‐1), and malondialdehyde (MDA, Cat# A003‐1‐2).

### 2.2 Aboratory Animal

#### 2.2.1 Animal Modelling

Twenty‐four adult male Sprague‐Dawley (SD) rats were housed under specific pathogen‐free (SPF) conditions for a 7‐day acclimatization period, with environmental parameters tightly regulated (temperature: 22°C ± 1°C; humidity: 55% ± 5%). The animals were then randomly assigned to four experimental groups (*n* = 6) and exposed to distinct therapeutic regimens over 14 consecutive days, as outlined in the experimental design (Table 1).

**Table 1:** Rat treatments.

groups	Mode of administration
Control	Daily intraperitoneal injection of 0.9% NaCl
Cd	Intraperitoneal injection of 1 mg/kg b.w. CdCl_2_
Cd + Nar	Intraperitoneally administered 1 mg/kg b.w. CdCl_2_ and 50 mg/kg b.w. Nar
Nar	Administer 50 mg/kg b.w. Nar

#### 2.2.2 Body Weight and Liver‐to‐Body Weight Ratio in Rats

Body weight was longitudinally tracked during the 14‐day experimental phase using calibrated digital scales. Initial measurements were recorded prior to treatment commencement (Day 0), followed by assessments at fixed intervals (Days 7 and 14). To ensure uniform Cd exposure (mg Cd/kg BW/day), dosages were modified in real‐time according to individual weight fluctuations. After a 12‐h fasting period (water provided ad libitum), rats were anesthetized with ether, weighed, and subjected to femoral artery blood collection prior to euthanasia via cervical dislocation. The abdominal cavity was surgically accessed through a midline incision, and liver tissues were excised, rinsed with saline, and surface moisture removed by blotting on absorbent filter paper.

### 2.3 Determination of Liver Biochemical and Antioxidant Indices

The ratio of liver tissue (0.1 g) to cold saline was 1:9 to prepare 10% (w/v) homogenate. The samples were kept on ice during mechanical grinding (60 s), and then centrifuged for 10 min (4°C) using a cryo‐centrifuge. Aliquots of the clarified supernatants were analyzed for oxidative stress markers (MDA) via spectrophotometric assays. AST, ALT, and GSH levels were quantified using a Tecan Infinite M Nano microplate reader (Switzerland) with manufacturer‐recommended protocols.

### 2.4 Pathological Tissue Sections

Histopathological analysis was conducted through hematoxylin and eosin (H&E) staining. Liver tissue fragments (5 × 5 × 3 mm^3^) were cut from the left lateral lobe and fixed in 4% paraformaldehyde for 48 h. Subsequent tissue processing included serial ethanol dehydration (70%−100%), xylene‐based clearing, and paraffin embedding using an automated tissue processor. The paraffin blocks were cut into 5 μM sections, dewaxed in xylene, and gradually rehydrated with ethanol (100%−70%). Tissue sections were stained with Mayer's hematoxylin and counterstained with eosin Y, with final morphological evaluation performed via brightfield microscopy.

### 2.5 Reverse Transcription Quantitative Real‐Time PCR (RT‐qPCR)

After grinding the tissue with a glass rod and adding chloroform, the tissue was shaken violently (15 s), centrifuged at 4°C, and the aqueous phase was separated. After that, the equal volume of isopropanol was added and the supernatant was discarded by centrifugation, and the RNA was washed twice with 75% alcohol. Finally, the RNA was dried and dissolved in an appropriate amount of non‐enzymatic water. The RNA was reversely transcribed into c DNA, and finally the expression of related genes was detected by fluorescence quantification.

### 2.6 Western Blot

Protein lysates were formulated by homogenizing 40 mg liver tissue in 400 μL RIPA buffer (Servicebio, G2002) supplemented with phosphatase inhibitor cocktail A (Beyotime, P1081) and protease inhibitor cocktail (Beyotime, P1010). The total protein concentration was determined by BCA method. The denatured protein samples were separated on 10% or 12% gel, and the membrane was transferred after electrophoresis. The cells were blocked with skimmed milk powder at room temperature for 2 h, and then incubated overnight with primary antibodies: GRP78, PERK and CHOP (stored at 4°C). After 3 times of TBST washing, the primary antibody was recovered and the secondary antibody was incubated for 1 h, and then the TBST was washed three times. The chemiluminescence signal was captured using an Omega Lum G imaging system (Aplegen, USA), and the band intensity was quantified by optical density analysis.

### 2.7 Statistical Analysis

SPSS 26 and Excel were used for data analysis to process the data of rat liver related indicators. One‐way analysis of variance (ANOVA) was used for SPASS 26 analysis and expressed as $\bar{x}$ ± SEM. *p* < 0.05 was considered statistically significant.

## 3 Result

### 3.1 Effect of Cd and Nar on Body Weight and Liver‐to‐Body Weight Ratio in Rats

**Figure 1:**
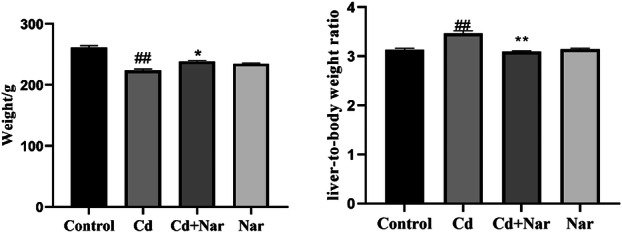
Body weight and liver‐to‐body weight ratio in rats, ^#^ indicates significant difference compared with control group (^#^ *p* < 0.05, ^##^ *p* < 0.01); * indicates significant difference compared with Cd group (*p < 0.05, **p < 0.01).

Figure 1 shows that compared with the control group, the body weight of the Cd group rats was significantly decreased (*p* < 0.01), and the liver coefficient (%) was significantly increased (*p* < 0.01). Compared with the Cd group, the body weight of the Cd + Nar group significantly increased (*p* < 0.05). The liver coefficient was significantly decreased (*p* < 0.01).

### 3.2 Effects of Cd and Nar on Liver Function

**Figure 2:**
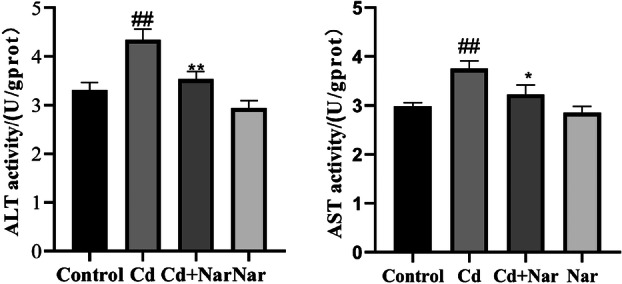
ALT versus AST activity. ^#^ Indicates significant difference compared with control group (^#^ *p* < 0.05, ^##^ *p* < 0.01); * indicates significant difference compared with Cd group (*p < 0.05, **p < 0.01).

Figure 2 indicates that, compared to the control group, the concentrations of ALT and AST in the Cd group were significantly increased (*p* < 0.01); in comparison to the Cd group, the ALT was significantly decreased (*p* < 0.01), and the AST was significantly decreased (*p* < 0.05). in the Cd + Nar group.

### 3.3 Effect of Cd and Nar on the Antioxidant Function of the Liver

**Figure 3:**
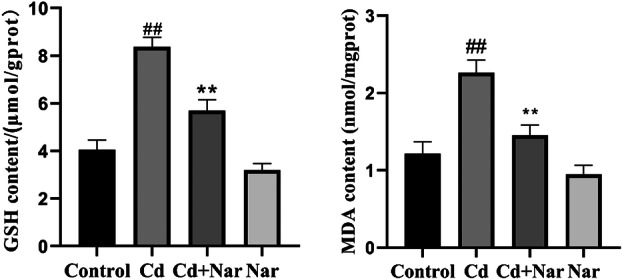
GSH and MDA content. ^#^ Indicates significant difference compared with control group (^#^ *p* < 0.05, ^##^ *p* < 0.01); * indicates significant difference compared with Cd group (*p < 0.05, **p < 0.01).

Figure 3 shows that compared with the control group, the contents of GSH and MDA in Cd group were significantly increased (*p* < 0.01). Compared with Cd group, GSH and MDA contents in Cd + Nar group were significantly decreased (*p* < 0.01).

### 3.4 Pathological Sections of the Liver

**Figure 4:**
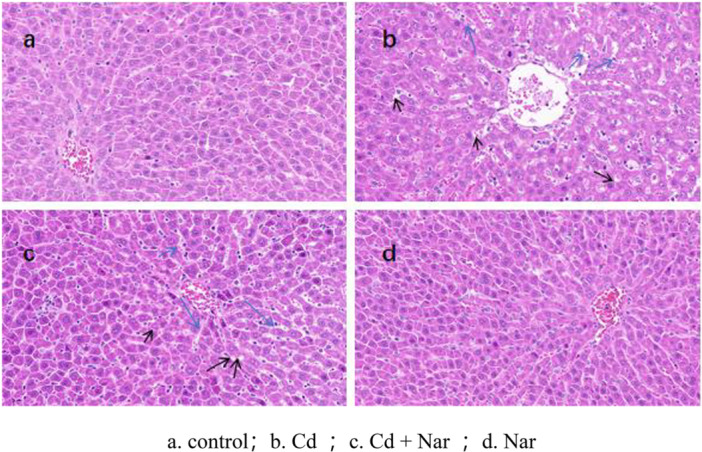
(a) control; (b) Cd; (c) Cd + Nar; (d) Nar. Pathological section of rat liver (x40) Blue arrows point to enlarged hepatic sinusoidal crevices; black arrows point to cytoplasmic vacuolization of hepatocytes.

Figure 4 shows that the structure of hepatic lobules was normal in the control group, and the central veins showed radioactive arrangement. Compared with the control group, the cytoplasm of hepatocytes in the Cd group was vacuolated, the gap of hepatic sinusoids was enlarged, the arrangement of hepatic cords was irregular, and there was no obvious boundary between hepatocytes, indicating that the cells were seriously damaged. Compared with the Cd group, the degree of vacuolization was smaller and the sinusoidal gap was relatively smaller in the Cd + Nar group.

### 3.5 Effect of Cd and Nar on the Expression of mRNA Related to the PERK Signaling Pathway

**Figure 5:**
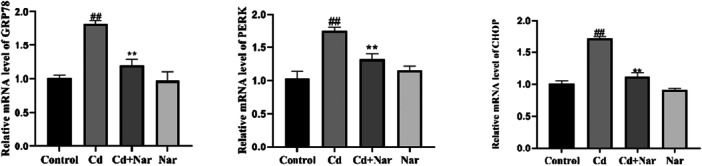
GRP78, PERK and CHOP gene mRNA content in rat liver. ^#^ Indicates significant difference compared with control group (^#^ *p* < 0.05, ^##^ *p* < 0.01); * indicates significant difference compared with Cd group (*p < 0.05, **p < 0.01).

As shown in Figure 5, compared with the control group, the expression of GRP78, PERK and CHOP genes in Cd group was significantly increased (*p* < 0.01), while there was no significant difference in Nar group. Compared with the Cd group, the expression of GRP78, PERK and CHOP mRNA in the Cd + Nar group was significantly decreased (*p* < 0.01).

### 3.6 Effects of Cd and Nar on the Expression of Proteins Related to the PERK Signaling Pathway

**Figure 6:**
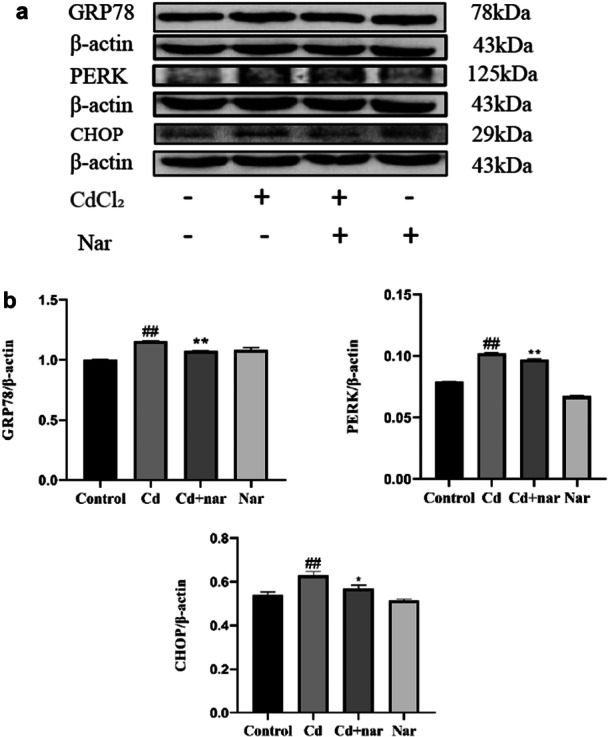
Effects of CdCl_2_ and Nar on ER stress‐related proteins. (a) Immunoblotting bands of GRP78, PERK and CHOP. (b) Western blot analysis of GRP78, PERK, CHOP. ^#^ Indicates significant difference compared with control group (^#^ *p* < 0.05, ^##^ *p* < 0.01); * indicates significant difference compared with Cd group (*p < 0.05, **p < 0.01).

As shown in Figure 6, compared with the control group, the expression of GRP78, PERK and CHOP protein in Cd group was significantly increased (*p* < 0.01). Compared with the Cd group, the expression of GRP78 and PERK protein in the Cd + Nar group was significantly decreased (*p* < 0.01). The expression of CHOP protein was significantly decreased (*p* < 0.05). There was no significant difference in Nar group compared with control group.

## 4 Discussion

Cd exposure exerts dose‐dependent effects on somatic growth parameters and hepatosomatic indices, as evidenced by progressive body weight loss and hepatic hypertrophy in exposed organisms [4]. Body mass serves as a composite biomarker reflecting systemic protein turnover, energy balance, and fluid homeostasis. Fluctuations in body mass trajectory correlate with multifactorial determinants including nutritional intake, metabolic dysregulation, and pathophysiological progression. Cd can cause serious toxic damage to the body of rats, and the degree of body damage can be obviously detected by the change of body weight. Elevated hepatosomatic indices typically reflect pathological processes including vascular congestion, interstitial edema, and compensatory cellular hypertrophy‐hallmarks of chemical‐induced hepatotoxicity. Xenobiotic exposure induces organ‐specific mass alterations through mechanisms ranging from inflammatory cell infiltration to parenchymal cell proliferation, quantifiable via organosomatic ratios. Jing et al. and Li et al. [8] reported a marked reduction in body weight among rats following 4‐week Cd administration, while Chen et al. and Xiaoming et al. [19] documented analogous manifestations in Cd‐exposed piglets, including unkempt fur and progressive emaciation. These findings collectively corroborate Cd's systemic toxicity, particularly its hepatotoxic effects. In the present study, co‐administration of Nar with Cd attenuated these adverse outcomes, evidenced by increased body weight and reduced hepatosomatic index in the Cd + Nar group compared to Cd‐treated rats. This suggests Nar's antioxidant properties may counteract Cd‐induced oxidative damage. Notably, Nar monotherapy exhibited no detectable toxicity [20], further supporting its potential as a protective adjuvant against heavy metal toxicity.

ALT and AST, key enzymes in amino acid metabolism, are recognized as sensitive serum biomarkers of hepatic injury due to their substantial intracellular accumulation in hepatocytes. As a primary target organ for Cd toxicity, the liver exhibits marked elevation of these enzymes following Cd‐induced cellular damage, reflecting compromised membrane integrity. This mechanistic correlation is substantiated by experimental models: Sengul et al. [2] demonstrated Nar's hepatoprotective efficacy through attenuated Cd‐induced ALT/AST release in rats. Similarly, Alfwuaires et al. [21] reported significantly elevated ALT and AST activities in Cd‐exposed cohorts compared to controls, whereas co‐treatment with Nar substantially reduced enzymatic levels. These collective findings confirm that pathological ALT/AST surges constitute a hallmark response to Cd‐mediated hepatotoxicity, which Nar supplementation effectively mitigates through antioxidant mechanisms. Experimental findings demonstrated pronounced elevations in serum AST and ALT levels among Cd‐exposed rats relative to the control cohort, confirming Cd‐induced hepatotoxicity. In contrast, co‐administration of Nar with Cd significantly attenuated these enzymatic markers compared to the Cd‐treated group, indicative of Nar's hepatoprotective capacity in mitigating Cd‐mediated liver injury.

GSH, a tripeptide synthesized from glutamate, cysteine, and glycine, serves as a critical component of endogenous antioxidant defenses. Conversely, malondialdehyde MDA, a terminal byproduct of lipid peroxidation cascades, functions as a biomarker for quantifying oxidative stress‐induced cellular damage. Cd may induce a sharp increase in ROS and trigger oxidative stress. Oxidative stress is considered to be one of the important mechanisms of cell damage caused by Cd. The affinity of Cd to sulfhydryl groups is stronger than that of other groups [20], so Cd entered into the body binds to sulfhydryl groups on proteins, leading to oxidative stress and anti‐oxidative damage [22]. It will eventually damage liver cells and is also a strong cause of ERS. Experimental evidence from Jing et al. and Li et al. [8] revealed significantly elevated MDA and diminished GSH levels in Cd‐exposed rats versus controls, a redox imbalance partially reversed by vitamin E co‐administration. Notably, Gao et al. [23] observed analogous MDA accumulation in HgCl_2_‐treated models, albeit with conflicting trends in GSH‐PX activity compared to the present Cd study. GSH could directly perform antioxidant effect, and GSH‐PX could indirectly perform antioxidant effect depending on GSH. This change may be because oxidative stress leads to tissue damage, and large amounts of antioxidants are required during tissue repair. This may further deplete GSH, and GSH‐PX is dependent on GSH to function, so GSH‐PX levels are also reduced. When rats were subjected to oxidative stress in this experiment, the body increased the synthesis and release of GSH in response to this stress, aiming to reduce the damage to cells. The reason for the two opposite results may be that the experimental animals were in different periods of oxidative stress, but whether the results are opposite or not, it can indicate that oxidative stress has occurred and the rats have been damaged. Thus Cd exacerbate oxidative stress in mice [24], aggravating liver damage, may be associated with Cd induced ROS produced [25]. Nar inhibits the production of unfolded protein to maintain the homeostasis of ER and protect the liver and body.

Studies have shown that Cd induces ERS, and when ERS occurs, the protein fails to fold as expected. Yue Zhu [4] found that ERS‐related mRNA and protein such as GRP78 and CHOP were significantly increased in Cd group compared with the control group, while their expression levels were significantly decreased after ASB (a kind of flavonoid compound) was added. According to the results of Pei‐Chao Gao [23], compared with the control group, the expression levels of mRNA such as GRP78, PERK and CHOP in HgCl_2_ group were significantly increased. Compared with HgCl_2_ group, the expression levels of GRP78, PERK and CHOP were significantly decreased in HgCl_2_ + Se group. Lulu Hou [26] used chicken Leyden cells treated with Se or Cd group to detect the expression of related genes and proteins in different time periods and found that the mRNA and protein expression levels of GRP78, PERK and CHOP in Cd group were increased in all time periods. The mRNA and protein expression of GRP78, PERK and CHOP decreased after Se treatment. Studies have found that GRP78, PERK, and CHOP are released when ERS occurs [27], resulting in the increase of the corresponding mRNA and protein content. Thus, the degree of damage can be judged by detecting the relevant content. Thus infer that Cd trigger ERS and Nar can inhibit the activation of PERK may can also through UPR ease ERS cause liver damage.

## Author Contributions

**Chengxiang Guo:** conceptualization, formal analysis, data curation, writing – original draft. **Hao Ling:** validation, formal analysis. **Mengmeng Gao:** data curation, formal analysis. **Yinan Hu:** methodology, formal analysis. **Jing Zhu:** formal analysis. **Jicang Wang:** conceptualization, funding acquisition, project administration, writing – original draft, writing – review and editing.

## Ethics Statement

All studies reported in the paper are conducted in an ethical and responsible manner and are in full compliance with all relevant experimental and legislative codes.

## Conflicts of Interest

The authors declare no conflicts of interest.

## Data Availability

The data presented in this study are available on reasonable request from the corresponding author.
